# Differential Cytotoxic Potential of *Acridocarpus orientalis* Leaf and Stem Extracts with the Ability to Induce Multiple Cell Death Pathways

**DOI:** 10.3390/molecules24213976

**Published:** 2019-11-03

**Authors:** Sameera Omar Mohammed Saeed Balhamar, Neena Gopinathan Panicker, Shaima Akhlaq, Mohammed Mansoor Qureshi, Waqar Ahmad, Najeeb Ur Rehman, Liaqat Ali, Ahmed Al-Harrasi, Javid Hussain, Farah Mustafa

**Affiliations:** 1 Department of Biochemistry, College of Medicine & Health Sciences, United Arab Emirates (UAE) University, Al Ain, P.O. Box 17666, UAE; mlt1987@hotmail.com (S.O.M.S.B.); mohammad.qureshi@uaeu.ac.ae (M.M.Q.); waqar.ahmad@uaeu.ac.ae (W.A.); 2 Natural and Medical Sciences Research Center, University of Nizwa, Sultanate of Oman, Nizwa, Oman; najeeb@unizwa.edu.om (N.U.R.); malikhejric@gmail.com (L.A.); aharrasi@unizwa.edu.om (A.A.-H.); 3 Department of Chemistry, University of Sargodha, Sub-Campus Mianwali, Punjab 42200, Pakistan; 4 Department of Biological Sciences & Chemistry, College of Arts and Sciences, University of Nizwa, Sultanate of Oman; javidhej@unizwa.edu.om

**Keywords:** *Acridocarpus orientalis*, traditional herbal medicine, phytochemicals, molecular mechanism, anticancer, apoptosis, necroptosis, autophagy, pharmacognosy, phytotherapy

## Abstract

This study systematically analyzed the anticancer potential of *Acridocarpus orientalis* (AO), a traditional medicinal plant of the Arabian Peninsula/East Africa known for its anti-inflammatory and pain relief properties. Tests of serial organic fractions from methanolic extracts of its leaves and stems revealed that only some fractions showed anti-proliferative potential with the dichloromethane fraction from leaves (AOD (L)) showing the most cytotoxic effect against both breast (MCF-7 and MDA-MB-231) and cervical (HeLa) cancer cell lines. The *n*-butanol fraction from the stems (AOB (S)), on the other hand, was more effective against cervical cancer cells and did not harm the normal cells. Further characterization of the mode of cell killing revealed that AOD (L) depended more on non-apoptotic pathways for its cytotoxicity in breast cancer cells, while it could activate some apoptosis and necroptosis in HeLa cells. The AOB (S) fraction could primarily activate apoptosis and some necroptosis in HeLa cells. Both fractions perturbed autophagy, but in a dissimilar manner. Thus, different parts of *A. orientalis* revealed variable potential to induce cell death in cancer cells via apoptotic and non-apoptotic pathways, making *A. orientalis* a valuable plant for the exploration of anticancer bioactive reagents, some of which may be protective for normal cells.

## 1. Introduction

*Acridocarpus orientalis* A. Juss (AO) is a medicinal plant of the family Malpighiaceae. Growing in rocky areas up to 1500 m, it is a rare plant species found mostly in the valleys and foothills of mountains in the United Arab Emirates (UAE) and Oman, especially surrounding Jabal Hafeet, Jabal Shams [1,2,3,4,5], and has also been reported from Somalia, growing at elevations between 100–700 m [6]. *A. orientalis* (regionally called qafas) is a small, highly branched shrub, having stems with hairy, and yellow flowers in clusters [1,6]. The flowers are bisexual, including male and female reproductive organs [7]. The young evergreen leaves are covered in reddish brown hair, which are lost upon growth, creating smooth leathery leaves with prominent veins [1,6,7]. The plant is known for its anti-inflammatory properties and is mostly used locally as a pain relief medicine. The crushed seeds form a crude extract and the oil produced from this plant are massaged onto the forehead and joints to relieve pain from chronic headaches, paralyzed limbs, and for muscle and tendon pain [1,4,8,9,10]. In Oman, it is used to treat the inflammation of mammary glands in cattle as well [9,10]. In Africa, several plant species in the genus *Acridocarpus* are used as medicine for gastrointestinal disorders, paralysis, and skin blisters (pemphigus) [11,12,13], and one species (*Acridocarpus chloropterus*) in particular has shown anti-parasitic activity against malarial, trypanosomal, and leishmanial pathogens [14]. Other than medicinal purposes, the plant is used as a source of yellow dye [3], while the reddish hair of the young leaves is used as a tanning agent [1].

The *Acridocarpus* genus has been shown to contain several phytochemicals, including the triterpenes: beta-sitosterol, stigmasterol, friedelin, oleanolic acid, and ursolic acid, and the flavonoids: apigenin, luteolin, vitexin, kaempferol, quercetin, and others [15,16]. *A. orientalis*, in particular, has been screened for its phytochemical content, revealing the presence of primary and secondary metabolites, including: carbohydrates, phenolic compounds, tannins, flavonoids, steroids, benzoquinones, and saponins [17,18]. The free radical scavenging activity estimated for *A. orientalis* fractions has shown that the antioxidant activity increases with increasing concentration of the extract [19]. In addition, significant levels of anti-lipoxygenase (anti-LOX) and anti-histone deacetylase (anti-HDAC) activities have also been reported in ethanolic extracts of aerial parts of this species harvested from the UAE and Oman [19]. Flavonoids isolated from methanolic extract of *A. orientalis* have shown antifungal, antioxidant, anti-lipid peroxidation, and cytotoxic activities [20]. Thus, *Acridocarpus* has great potential as an important source of bioactive compounds for drug discovery as well as directly as a treatment for various illnesses such as arthritis, atherosclerosis, stroke, diabetes, neurological disorders, and cancers [20,21].

Despite a wealth of information regarding the phytochemical composition of this plant, not much is known about its anticancer potential. Thus, we carried out a systematic analysis of the anticancer potential of leaves and stems of *A. orientalis* by testing sequential organic fractions of their methanolic extracts that had earlier shown to have some cytotoxic potential [22]. After confirming their anti-proliferation potential in various human breast and cervical cancer cell lines, the mechanism of action of their fractions was explored by characterizing their ability to induce apoptosis, an important cell death pathway activated by anticancer agents [23,24]. 

## 2. Results

### 2.1. Effect of Different Leaf (L) and Stem (S) Crude Fractions of A. Orientalis on Cancer Cell Proliferation

To test the anticancer potential of *A. orientalis,* the different extracts and fractions of the leaves and stems of the plant were screened for their effects on cancer cell viability using the MCF-7 (breast [25]) and HeLa (cervical [26]) cancer cells treated for 24 and 72 h. We chose cancer cell lines from two different cancer types to ensure that we did not miss the therapeutic potential of the plant which can be effective in one cell type, but not another. The organic extract/fractions were dissolved in DMSO, while the aqueous fractions were dissolved in water and tested in MTT assays using 50, 125, and 250 µg/mL concentrations. Table 1 shows the results of the assay where the extracts/fractions that did not show any effect on cell viability were marked with a cross, while those that resulted in >20% cell death were shown with a check mark. As can be seen, 1) most (8/12 or ~67%) of the extracts/fractions tested were not effective for killing cancer cells, and 2) of the four effective fractions (AOD (L), AOEA (L), AOD (S), and AOB (S); see Table 1 for full fraction names), most (3 out of 4) were effective at killing only HeLa and not MCF-7 cells (Table 1). Interestingly, the dichloromethane solvent was the best at extracting anticancer activity from the methanolic extract of both stems and leaves. This activity was variable since while AOD (L) showed anti-proliferative effect for both breast and cervical cancer cells, AOD (S) was more effective for killing HeLa cells. The other two fractions, AOEA (L) and AOB (S) were effective at killing the HeLa cells only (Table 1). This shows that dichloromethane is a good solvent for extracting anticancer biomolecules. Based on these results, we selected AOD (L) and AOB (S) as representative fractions of the leaves and stems with different potentials to kill the two types of cancer cells and characterized these fractions further for their anticancer potential.

### 2.2. AOD (L) and AOB (S) Reveal Different Anticancer Potentials 

**Figure 1:**
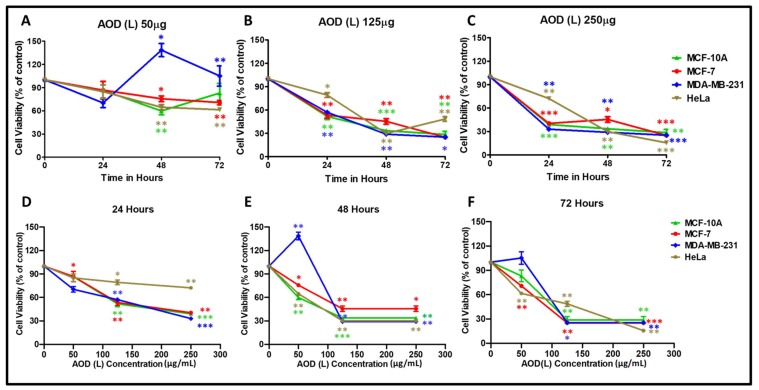
Cytotoxic effects of *A. orientalis* dichloromethane leaf fraction (AOD (L)) using the MTT assay on MCF-10A, MCF-7, MDA-MB-231, and HeLa cells in a dose- (**A**–**C**) and time- (**D**–**F**) dependent manner after normalizing to the effects of DMSO solvent used to dissolve the extract. *indicates statistically significant differences between the DMSO- and AOD (L)-treated samples (* *p* < 0.05; ** *p* < 0.01 but >0.001; *** *p* < 0.001).

**Figure 2:**
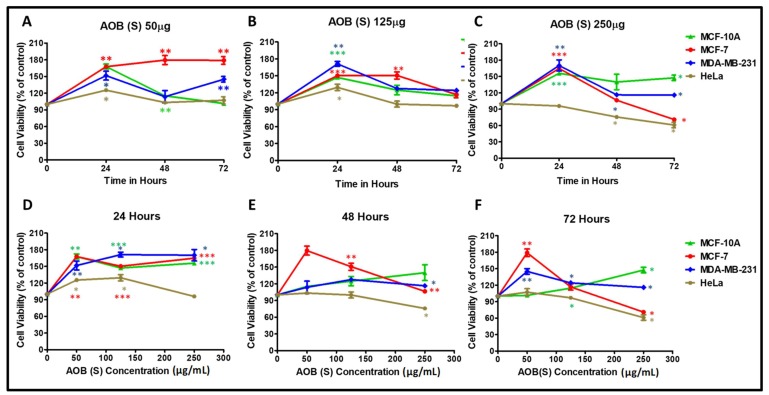
Cytotoxic effects of *A. orientalis* dichloromethane leaf fraction (AOB (S)) using the MTT assay on MCF-10A, MCF-7, MDA-MB-231, and HeLa cells in a dose- (**A**–**C**) and time- (**D**–**F**) dependent manner after normalizing to the effects of DMSO solvent used to dissolve the extract. *indicates statistically significant differences between the DMSO- and AOD (L)-treated samples (* *p* < 0.05; ** *p* < 0.01 but >0.001; *** *p* < 0.001).

To characterize these fractions further, AOD (L) and AOB (S) were retested on a larger panel of cell lines that included: the normal breast epithelial cell line (MCF-10A) [27] and two breast cancer cell lines, the hormone responsive (MCF-7) cells [25], the hard to treat triple hormone receptor-negative cell line (MDA-MB-231) [28], and the cervical cancer cell line (HeLa) [26]. The proliferative response of the treated cells was normalized to that of the corresponding DMSO controls and plotted in a dose- and time-dependent manner (Figure 1 and Figure 2). As can be seen, AOD (L) was efficient at inducing death in all the four cell lines in a statistically significant manner, leading to 70–80% death of cells by 72 h at the highest dose tested (250 µg/mL) (Figure 1C,F). The MD-MB-231 cells were less sensitive to AOD (L)-induced killing at the lower dose of 50 µg/mL and in fact resulted in their proliferation, but the 125 µg/mL dose was sufficient to kill these cells effectively (Figure 1A vs. Figure 1B). 

The time course of killing revealed a similar trend of cell death among the four cell lines, except for a slight delay observed with the HeLa cells at the 24-h time point (Figure 1D–F). Calculation of the dose of AOD (L) that caused 50% cell death (IC_50_) for each cell line revealed the following IC_50_ values: 83.4 µg/mL for HeLa, 98.5 µg/mL for MCF-7, 167.4 µg/mL for MCF-10A, and 201.5 µg/mL for MDA-MB-231, respectively. These results clearly reveal that the leaf fraction from *A. orientalis* has strong cytotoxic activity for three types of cancer cells, similar to what was observed earlier (Table 1). Unfortunately, it also shows that the potential of AOD (L) to kill the cancer cells was similar to that observed for the normal mammary epithelial cells.

Test of the AOB (S) fraction on the four cell lines revealed that in sharp contrast to the leaf fraction, the AOB (S) fraction demonstrated much less cytotoxicity for cancer cells (Figure 2). For instance, it induced killing of MCF-7 and HeLa cells by ~25–30% (Figure 2C,F), as observed earlier (Table 1); however, in contrast to AOD (L), AOB (S) led to the proliferation of MDA-MB-231 cells and that of normal MCF-10A cells as well, in a statistically-significant manner (Figure 2C,F).

Together, test of the organic extracts/fractions of the leaves and stems of *A. orientalis* reveals that leaves of the plant have potent anticancer activity for cancer cells, but they also harm the normal cells, while the stem is enriched in compounds that have anticancer properties against two cancer cell lines (one breast cancer and another cervical cancer), activities that do not harm the normal cells.

### 2.3. AOB (S) can Activate Significant Caspase Activity in HeLa Cells 

**Figure 3:**
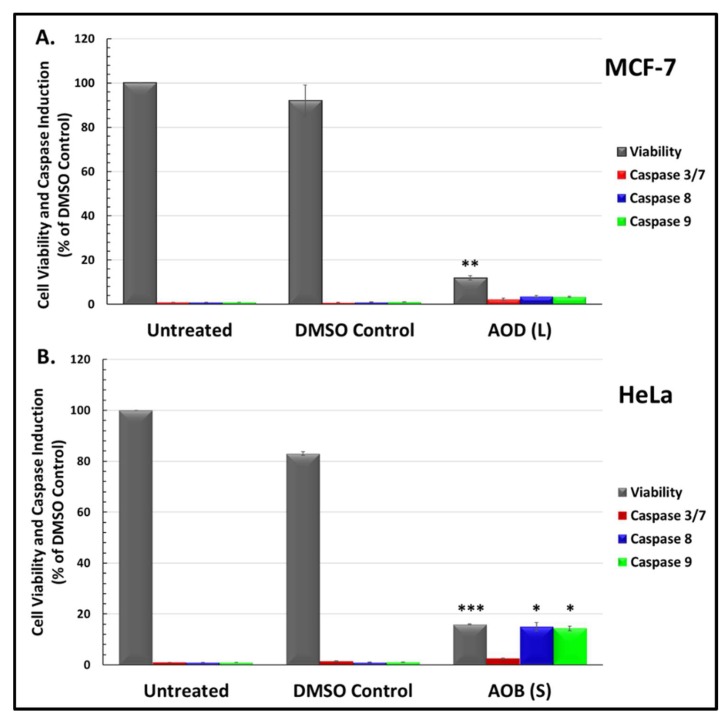
Viability and caspase activity of *A. orientalis*: (**A**) dichloromethane leaf fraction (AOD (L)) in MCF-7 cells, and (**B**) n-butanol stem fraction (AOB (S)) in HeLa cells treated with 250 µg/mL for 48 h. *, statistically significant differences between the control and treated samples (* *p* < 0.05; ** *p* < 0.01 but >0.001; *** *p* < 0.001). DMSO C, DMSO control having same amount of DMSO as the test treated sample.

To explore the potential mechanism of action of the effective leaf and stem fractions, their ability to induce apoptosis was explored, being the most widely induced type of cell death pathway observed in cancer [23,24]. To this end, the Promega Caspase-Glo^®^assays were used to detect activation of caspase enzymes 3/7, 8, and 9, that are classically activated upon apoptosis [23]. Since AOD (L) was equally effective in both breast and cervical cancer cell lines (Figure 2), MCF-7 cells were selected to test its efficacy in activating the caspases, while HeLa cells were selected for the treatment with AOB (S) as they were the most significantly affected cell line. All results obtained for the induction of caspases were normalized to the number of viable cells in culture (Figure 3). 

Test of AOD (L) on MCF-7 cells revealed that it was able to induce >80% cell death in culture, as demonstrated earlier by the MTT assay (Figure 1). Despite the significant cytotoxicity, it was not efficient at inducing caspase activity, as observed in the Glo assay (Figure 3A). The enzymatic activity for caspase 3/7, 8, and 9 was detectable, but not statistically significant, even though highly significant levels of caspase enzymatic activity for all these caspases could be detected at the same time in the same assay with an essential oil from *Boswellia sacra*, that has potent apoptotic activity (data not shown) [29,30]. A repeat of the experiment essentially gave the same results, suggesting that the primary mode of cell death induced by AOD (L) in MCF-7 cells is other than apoptosis. 

**Figure 4:**
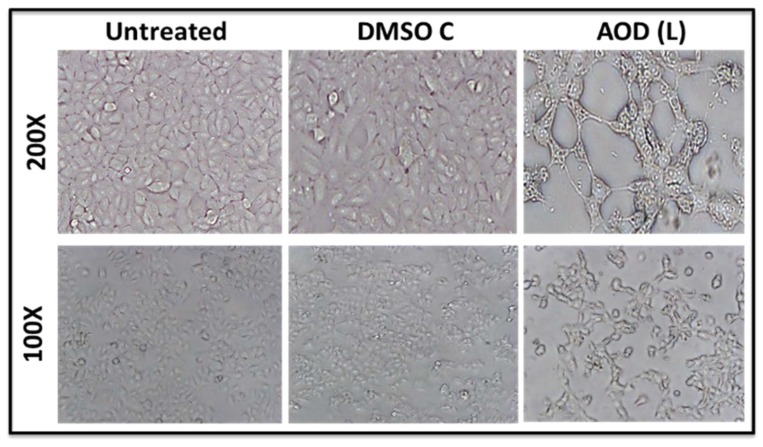
Photomicrographs of MCF-7 cells treated with 250 µg/mL of *A. orientalis* dichloromethane leaf fraction (AOD (L)), 30 h post treatment. DMSO C, DMSO control having the same amount of DMSO as the test sample.

Morphological analysis of MCF-7 cells treated with AOD (L) under a light microscope revealed a lack of cell rounding, shrinkage, and detachment from other cells, all characteristic of cells undergoing apoptosis (Figure 4); rather, the treated samples revealed morphological changes reminiscent of autophagy—a marked appearance of vacuoles in the cell cytoplasm and loss of cells from the monolayer without cell rounding (Figure 4) [31,32].

Test of the AOB (S) fraction on HeLa cells in the Glo assays revealed that it was much better than AOD (L) in inducing caspases (Figure 3B). Among the four caspases tested, it was able to activate caspases 8 and 9 in a statistically significant manner, while activation of caspase 3/7 was detectable, but not statistically significant (Figure 3B). This suggests that AOB (S) fraction has compounds that can induce apoptosis.

### 2.4. Both AOD (L) and AOB (S) can Lead to PARP Cleavage 

**Figure 5:**
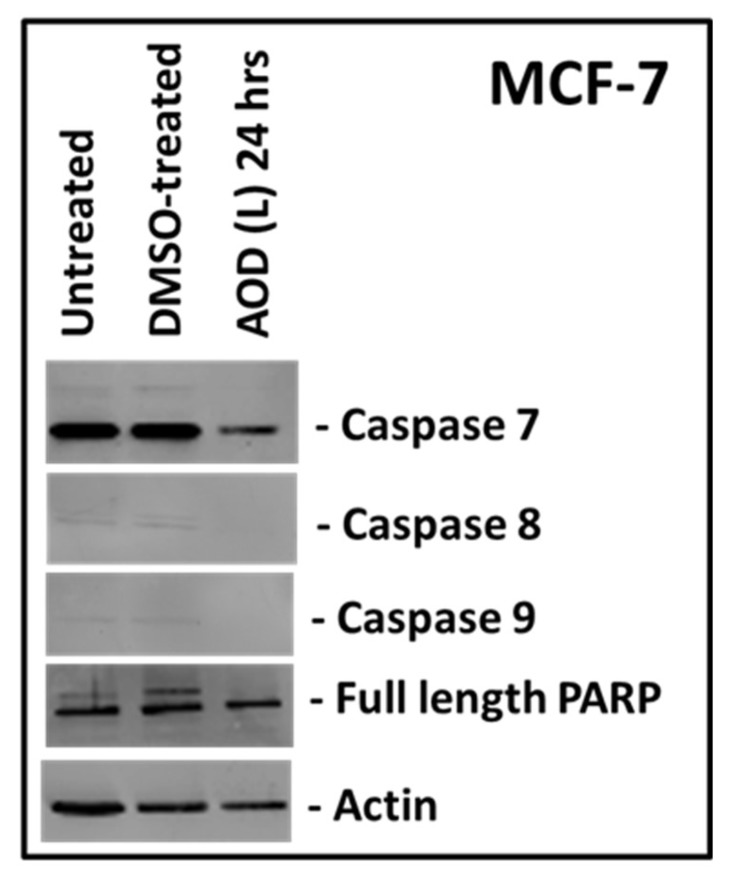
Western blot analysis of MCF-7 cells treated with *A. orientalis* dichloromethane leaf fraction (AOD (L)) at 250 µg/mL and tested for the activation of caspases 7, 8 and 9, and poly ADB ribose polymerase (PARP). Actin antibody was used as a loading control. DMSO-treated, DMSO control having the same amount of DMSO as the test sample.

To confirm these results, we tested the ability of AOD (L) to induce cleavage of apoptosis-specific proteins via western blot analysis. MCF-7 cells were treated with AOD (L), and cell lysates were prepared, and tested for the expression and cleavage of various caspases and poly ADP-ribose polymerase (PARP), the two major hallmarks of a cell undergoing apoptosis (Figure 5) [23]. As can be seen, cleavage of none of the three caspases tested (caspase 7, 8, or 9) could be detected; however, some loss of the pro form of these caspases could be detected as well as full-length PARP at 24 h post treatment compared to actin control (Figure 5). This suggests that AOD (L) may have a slight potential to activate caspases in MCF-7 cells that one cannot rule out since crude fractions are being tested where concentration of the active compounds could be very low. 

**Figure 6:**
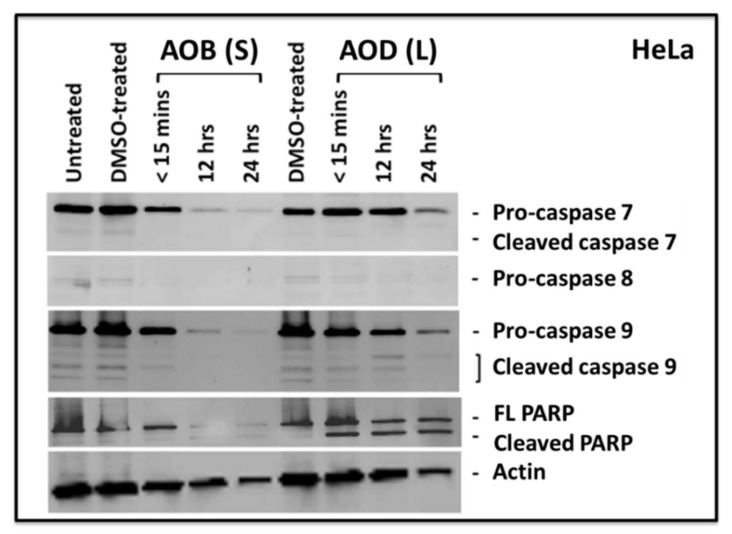
Western blots of HeLa cells treated with 250 µg/mL of *A. orientalis n*-butanol stem (AOB (S)) or dichloromethane (AOD (L)) leaf fractions for the indicated time points and tested for the activation of caspase 7, 8, and 9, as well as poly ADB ribose polymerase (PARP). FL, full length. DMSO-treated, DMSO control having the same amount of DMSO as the test sample.

Since caspase 8 and 9 were difficult to detect in MCF-7 cells, while AOD (L) was equally cytotoxic in HeLa cells, therefore we decided to test HeLa cells treated with either AOD (L) or AOB (S) to analyze their ability to induce activation of apoptotic proteins. As can be seen in Figure 6, both fractions were capable of inducing cleavage of PARP, while a time-dependent disappearance of the pro forms of caspase 7, 8, and 9 could be observed (Figure 6). Some effect of the tested fractions on actin degradation could be observed at 24 h, suggesting late stages of apoptosis when actin degradation has been observed [29,33]. This is supported by the observation that PARP cleavage could be detected at the earlier time points (<15 min and 12 h) in the absence of effects on actin integrity (Figure 6). Furthermore, the inability to detect caspase cleavage products despite clearly detecting PARP cleavage at multiple time points and with both fractions could be due to the well-known unstable nature of the cleaved caspase products and the inability of the antibody to detect them properly [34]. This assertion is further supported by our earlier observation that we could detect statistically significant levels of caspase 8 and 9 enzymatic activity in our caspase Glo assays (Figure 4). 

### 2.5. Test of the Potential of AOD (L) and AOB (S) to Induce Early Signs of Apoptosis in HeLa Cells 

**Figure 7:**
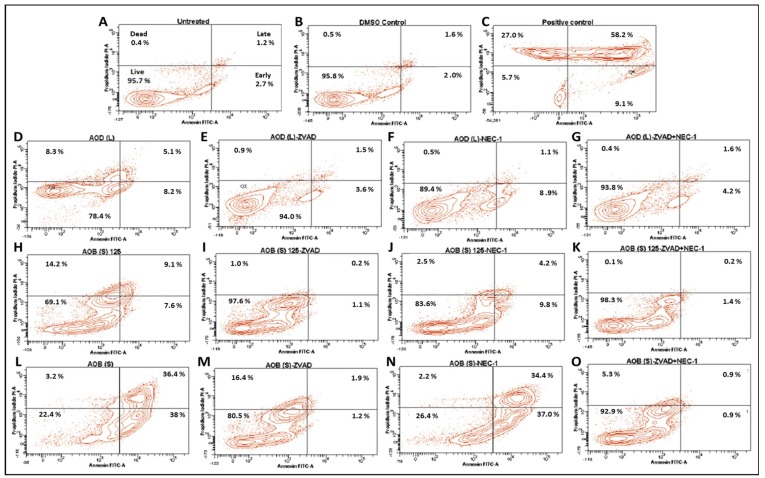
Annexin V-FITC/propidium iodide (PI) analysis of HeLa cells treated with 250 µg/mL of the *A. orientalis* dichloromethane leaf (AOD (L)) fraction or 125 and 250 µg/mL n-butanol stem (AOB (S)) fraction for 24 h. Samples: (**A**) Untreated; (**B**) DMSO control; (**C**) Positive control-treated; (**D**) 250 µg/mL AOD (L)-treated; (**E**) 250 µg/mL AOD (L)-treated cells pretreated with ZVAD, or (**F**) NEC-1, or (**G**) both; (**H**) 125 µg/mL AOB (S)-treated; (**I**) 125 µg/mL AOB (S)-treated cells pretreated with ZVAD, or (**J**) NEC-1, or (**K**) both; (**L**) 250 µg/mL AOB (S)-treated; (**M**) 250 µg/mL AOB (S)-treated cells pretreated with ZVAD, or (**N**) NEC-1, or (**O**) both. ZVAD = Z-VAD-FMK; NEC-1, Necrostatin 1. The DMSO Control sample contains the same amount of DMSO as in the 250 µg/mL sample of the treatment.

**Figure 8:**
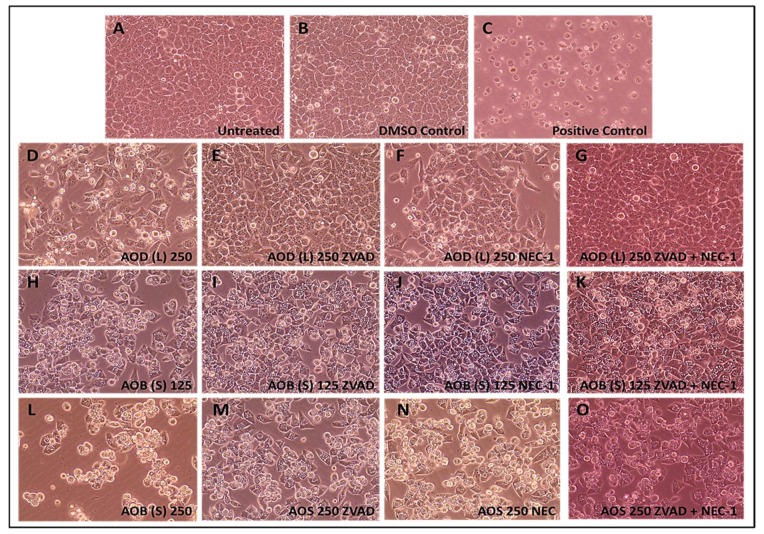
Photomicrographs of HeLa cells treated with 250 µg/mL of the *A. orientalis* dichloromethane leaf (AOD (L)) fraction or 125 and 250 µg/mL n-butanol stem (AOB (S)) fraction for 24 h. Samples: (**A**) Untreated; (**B**) DMSO control; (**C**) Positive control-treated; (**D**) 250 µg/mL AOD (L)-treated; (**E**) 250 µg/mL AOD (L)-treated cells pretreated with ZVAD, or (**F**) NEC-1, or (**G**) both; (**H**) 125 µg/mL AOB (S)-treated; (**I**) 125 µg/mL AOB (S)-treated cells pretreated with ZVAD, or (**J**) NEC-1, or (**K**) both; (**L**) 250 µg/mL AOB (S)-treated; (**M**) 250 µg/mL AOB (S)-treated cells pretreated with ZVAD, or (**N**) NEC-1, or (**O**) both. ZVAD = Z-VAD-FMK; NEC-1, Necrostatin 1. The DMSO Control sample contains the same amount of DMSO as in the 250 µg/mL sample of the treatment. Magnification: 400×.

To follow up on these observations, we tested the ability of AOD (L) and AOB (S) to induce early signs of apoptosis which are manifested by the inversion of a cell’s phospholipid bilayer, exposing the phospholipids found on the inner surface of the plasma membrane such as phosphatidylserine as well as proteins [23,35]. Thus, the annexin V/propidium iodide (AV/PI) assay was used to study this phenomenon (Figure 7 and Figure 8). As mentioned earlier, treatment of HeLa cells with the essential oil from *Boswellia sacra* (a positive control [29,30]) led to shifting of the treated cells in the AV(+)/PI(−) quadrant, indicative of early apoptosis (9.1%), and AV(+)/PI(+) quadrant, indicative of late apoptosis (58.2%) compared to the untreated- and DMSO-treated cells (Figure 7A–C). Treatment of cells with AOD (L) led to a more attenuated, but clearly observable effect on apoptosis, shifting 8.2% of the HeLa cells into the early (AV(+) only quadrant) and 5.1% of the cells into the late phase of apoptosis (AV(+)/PI(+) quadrant) (Figure 7D). Pre-treatment of the AOD (L)-treated cells with the apoptosis inhibitor, Z-VAK-FMK, was essentially able to reverse this phenomenon (Figure 7E), confirming that AOD (L) could induce apoptosis in HeLa cells, as observed by the activation of various caspases and PARP by western blot analysis (Figure 5 and Figure 6). 

To determine whether AOD (L) had the ability to induce other cell death pathways such as necroptosis that shares characteristics of both apoptosis and necrosis [36], HeLa cells were pretreated with a necroptosis inhibitor, necrostatin-1 (NEC-1) either alone or in the presence of Z-VAD-FMK prior to treatment with AOD (L). As can be seen from Figure 7F, pretreatment with NEC-1 led to reduction of cells in the late apoptosis stage from 5.1% to 1.1%, consistent with increase in the overall survival rate of the cells from 78.4 to 89.4% (compare Figure 7F vs. Figure 7D). However, the percent cells in the early apoptotic stage remained the same, suggesting partial reversal. Double treatment with Z-VAD-FMK and NEC-1 reversed AOD (L)-induced cell death to essentially the same level as DMSO-treated cells, increasing cell viability from 78.4 to 93.8%. This data suggests that AOD (L) can induce both apoptosis and another cell death pathway, necroptosis, in HeLa cells [36].

To investigate the effect of AOB (S) on HeLa cells, two different concentrations of the fraction were tested, 125 and 250 µg/mL, to ensure that early apoptotic events were not missed since AOB (S) had a more potent effect on apoptosis (Figure 3 and Figure 7). Treatment of HeLa cells with 125 µg/mL of AOB (S) revealed that both early (7.6%) and late (9.1%) apoptosis could be observed at nearly the same levels as treatment with 250 µg/mL of AOD (L); i.e., 8.2% and 5.1%, respectively. This increased to 38% and 36.4%, respectively, with doubling the dose of AOB (S) (compare Figure 7H vs. Figure 7L), confirming our earlier assessment that AOB (S) had a better potential of inducing apoptosis than AOD (L). Pre-treatment with Z-VAD-FMK was essentially able to revere the cell death incurred by 125 µg/mL dose of AOB (S) (Figure 7I), while pre-treatment with NEC-1 was only partially able to reverse the cell death observed by 125 µg/mL of AOB (S) (Figure 7J), similar to AOD (L) (Figure 7F), suggesting that AOB (S) could activate necroptosis as well, though to a lower extent. This was confirmed by dual treatment with the two inhibitors, which was able to completely reverse the AOB (S)-induced cell death at 125 µg/mL dose (Figure 7K). On the other hand, Z-VAD-FMK was only partially able to reverse the effects of the 250 µg/mL dose of AOB (S), with 16.4% of the cells remaining in the PI(+) only quadrant that signifies “dead cells” that can take up the PI dye without showing signs of membrane inversion (Figure 7M). Interestingly, NEC-1 had no effect on AOB (S)-induced cell death at 250 µg/mL, despite the fact that it could partially reverse its effect on cell death at the lower dose (Figure 7N). Dual treatment with the two inhibitors was able to restore cell viability to nearly the DMSO-treatment level (compared Figure 7O with Figure 7B). These data suggest that AOB (S) can induce not only apoptosis, but at lower doses, can activate necroptosis, similar to AOD (L) at higher doses. However, at higher doses, AOB (S) primarily activates apoptosis. Whether higher doses of Z-VAD-FMK can overcome this effect remains to be tested. 

Light microscopic analysis of the AOD (L)- and AOB (S)-treated cells supported these results. Treatment of HeLa cells with the positive control clearly led to apoptosis with most of the cells in culture rounding up and detaching from the plate (Figure 8A). A similar phenotype was observed with the treatment of HeLa cells with AOD (L), but to a lower extent (Figure 8D) which was completely or partially reversed by Z-VAD-FMK (Figure 8E) and NEC-1 (Figure 8F), respectively. Microscopic analysis of the AOB (S)-treated cells revealed a similar rounding of cells and their detachment from the plate as in AOD (L) except that the cells came off in bunches and the degree of cell loss was greater in the higher dose of AOB (S)-treated cells (Figure 8H vs. Figure 8L). This loss of cells was inhibited/decreased by treatment with Z-VAD-FMK (Figure 8I,M) or NEC-1 (Figure 8J,N), similar to AOD (L). Dual treatment with the inhibitors resulted in restoration of cell numbers in the 125 μg/mL-treated sample (Figure 8K), but not the 250 ug/mL-treated sample (Figure 8O), as observed upon FACS (Figure 7O), revealing loss of about 5% of the cells. These results support the observation that both AOD (L) and AOB (S) have compounds that can induce apoptosis in HeLa cells.

### 2.6. AOD (L) and AOB (S) Perturb Autophagy 

**Figure 9:**
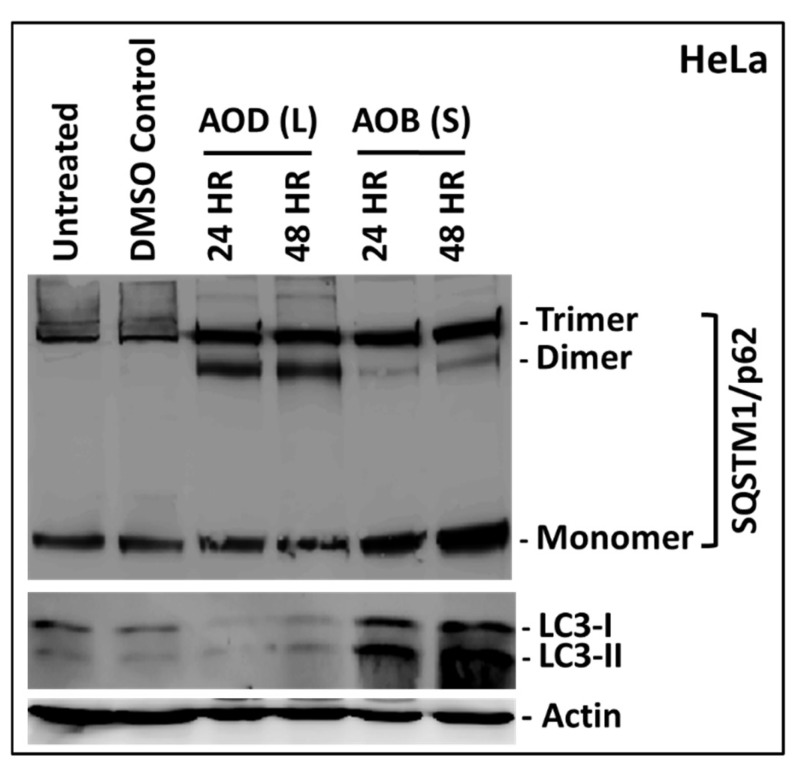
Western blot analysis of HeLa cells treated with 250 µg/mL of *A. orientalis* dichloromethane leaf (AOD (L)) or *n*-butanol stem fractions (AOB (S)) for 24 and 48 h. Actin antibody was used as a loading control. Different oligomeric forms of SQSTM1 were observed on the gel, that have been indicated on the side. DMSO C, DMSO control having same amount of DMSO as the test treated sample.

Since the two fractions had the potential to induce apoptosis as well as necroptosis, we next explored whether these fractions had the ability to induce autophagy, a type II programmed cell death pathway that is activated by anticancer agents [37,38,39]. As can be seen from Figure 9, treatment of HeLa cells with AOD (L) for 24 h was sufficient to induce the expression of both dimeric and trimeric forms of SQSTIM1 over and above the ones present in the untreated and DMSO-treated cells (first two lanes in Figure 9). These forms have been reported in literature recently that arise as a result of oxidative stress on the cell, causing oligomerization of the protein [40]. Treatment with AOB (S) also affected SQSTM1, but resulted in further induction of the monomeric and trimeric forms, at the expense of dimeric forms (Figure 9). This pattern of induction and oligomerization of SQSTM1 by AOB (S) was clearly different from the one observed for AOB (L), supporting our observation that AOD (L) and AOB (S) affect HeLa cells differently. Analysis of LC3 by western blot further confirmed these observations since AOD (L) led to degradation of LC3-I, while treatment with AOB (S) led to an increase in both the LC3-I and LC3-II forms of the protein (Figure 9, bottom panel). These results suggest that both AOD (L) and AOB (S) fractions have bioactive compounds that perturb autophagy. The mechanism of their action remains to be explored in future studies. 

## 3. Discussion

This study was conducted to characterize the anticancer potential of the medicinal plant *A. orientalis* for which both the leaves and stem fractions were tested separately. Overall, our results reveal that *A. orientalis* has significant anticancer potential against human cancer cell lines and most of the activity resides primarily within its leaves. Furthermore, it has the potential to induce apoptosis, necroptosis, and autophagy in human cervical cancer, while probably only autophagy in breast cancer cells. Thus, our results demonstrate that *A. orientalis* is a good candidate for the exploration of anti-neoplastic agents from nature with variable bioactivities against different types of cancers.

More specifically, a total of twelve crude organic and aqueous fractions were initially screened to determine which fraction had potential anticancer activity for two human cancer cell lines: MCF-7 and HeLa (Table 1). The main conclusions from this screening were: 1) only a limited number of fractions showed significant (>20%) anti-proliferation activity; 2) dichloromethane was the most effective solvent for the extraction of anticancer activities from amongst the tested solvents; and 3) there were far fewer fractions effective on breast cancer cells (1 of 12) compared to cervical cancer cells (4 of 12) (Table 1). This is similar to what has been found earlier and makes our observation especially important since finding anticancer activity against breast cancer cells is harder to find in large-scale screening of natural plants than other cancers. For example, only 11 out of 1000 extracts were observed to be effective for MCF-7 cells prepared from 351 plants from the Brazilian rain forests [41], while only ~10 extracts out of 7500 extracts prepared from South African plants showed moderate activity against MCF-7 cells [42]. Overall, these data suggest that phytochemicals from *A. orientalis* have anticancer potential against breast cancer cells, but this potential is more potent against cervical than breast cancer cells. 

Further screening of the two selected fractions, AOD (L) and AOB (S) revealed that most of the cytotoxic activity of the plant was observed in its leaves since the AOD (L) fraction could induce cell death significantly in all the four cell lines tested and to a much higher extent than AOB (S) (Figure 1 and Figure 2). AOB (S), on the other hand, had a more selective cytotoxicity profile with the ability to induce death of MCF-7 and HeLa cells, but not of the triple negative MDA-MB-231 (Figure 2). Furthermore, and more importantly, it had no cytotoxic effect on the normal MCF-10A breast cells (Figure 1). These results reveal that both the stem and leaves have anticancer compounds that are distinct from each other since they have different specificities. It is well-known that essentially any part of a plant may potentially contain anticancer activity, including flowers, fruit, stem, leaves, roots, seeds, bark, rhizomes, buds, sprouts, and gum, though leaves seem to be most frequently (~27%) associated with it [43]. This probably is due to the higher frequency of their being tested for anticancer activity.

Interestingly, we observed that lower doses of AOD (L) (50 µg/mL) lead to the proliferation of MD-MB-231 cells compared to the 125 µg/mL dose, which was sufficient to kill these cells effectively (Figure 1A vs. Figure 1B). We have observed a similar effect on cell proliferation at the lower doses before with other crude plant extracts at times ([29] and unpublished observations). We feel that it could be due to the reported (though less known) artefact of the MTT assay where certain compounds in the extract enhance the mitochondrial reductase activity rather than an actual effect on cell proliferation. Such an effect can be separated from a real effect on cell proliferation by its transient nature and is lost upon higher doses of the extract, as was the case in Figure 1 [44,45,46]. 

Analysis of the mechanism of cell death being activated by these fractions revealed that both AOD (L) and AOB (S) could induce apoptosis in HeLa cells, as observed by their ability to induce PARP cleavage. PARP is one of the enzymes that is cleaved during the execution phase of apoptosis by activated caspases [23]. However, AOB (S) was more effective at inducing PARP cleavage than AOD (L) as a near complete degradation of both cleaved and full length PARP was apparent within 12 h of treatment with AOB (S) compared to AOD (L) (Figure 6). Furthermore, despite high cytotoxicity for breast cancer cells, AOD (L) was less efficient at activating apoptosis in MCF-7 cells (Figure 3, Figure 4, Figure 5 and Figure 6). This was in comparison to AOB (S) that had moderate cytotoxic effects on HeLa cells and yet was able to induce caspase enzyme activity better than AOD (L) (Figure 5, Figure 6, Figure 7 and Figure 8). This observation suggests that apoptosis may be the major mechanism of action of AOB (S) in HeLa cells, while even though AOD (L) could induce apoptosis in HeLa cells, its primary mechanism of cell death induction in MCF-7 cells is non-apoptotic in nature, such as perhaps autophagy, a cell death mechanism that should be investigated further (Figure 9). Comparison of the morphological appearance of treated cells supports this observation as cells with more “apoptotic” cell morphology were observed in AOB (S)-treated cells consisting of cell shrinkage, rounding up, and coming off the plate (Figure 8), while AOD (L)-treated cells revealed a distinct morphology with increased vacuolization of the cytoplasm, a feature more reminiscent of autophagy (Figure 4). This is despite the fact that AOD (L) had a higher cytotoxic potential than AOB (S) (Figure 2 vs. Figure 3). This was further confirmed by the activation of caspase 8 and 9 activities by AOB (S) compared to AOD (L) (Figure 3). 

Many natural compounds with anticancer activity have been shown to use autophagy as their mechanism of action in cancer cells [39,40]. Autophagy is an innate cellular mechanism responsible for maintaining cellular homeostasis by recycling and eliminating damaged cellular components such as organelles and proteins using the lysosomal pathway [37,38,39]. Due to this important role, autophagy has been proposed to suppress cell transformation in healthy cells and its temporary loss may be accompanied by cell transformation [47]. Thus, we analyzed the ability of AOD (L) and AOB (S) to interact with autophagy markers, LC3 and SQSTM1. LC3 is a soluble cytosolic protein that is cleaved by cellular protease upon induction of autophagy into LC3-I and subsequently recruited onto the developing membranous autophagophore in the cytosol via lipidation, creating LC3-II. This leads to a change in its size and location in the cell [37,38]. SQSTM1, on the other hand, is the main cellular protein (also called autophagy receptor) that recruits the ubiquitinated bulk protein and organelle cargo destined for destruction to the autophagophore-lysosome by binding to LC3-II [37,38]. Fluctuations in the activation, recruitment, and/or cleavage of these proteins suggests perturbation in the process of autophagy [48,49,50]. Our data revealed that both fractions could perturb autophagic activity, although differently (Figure 9); while AOD (L) led to degradation of LC3-I, AOB (S) led to its accumulation in HeLa cells. This suggests that AOD (L) could be activating autophagy in HeLa cells, while AOB (S) may be inhibiting it, as observed with the accumulation of SQSTM1 in AOB (S)-treated HeLa samples (Figure 9). Activation of autophagy in HeLa cells by AOD (L) suggests that it may be the primary mode of inducing cell death in MCF-7 cells also where high levels of cell death could be observed in the absence of caspase activation (Figure 3), but that remains to be investigated further. 

It is increasingly being observed that autophagy can either induce cell death along with apoptosis or induce cell survival by inhibiting apoptosis. Moreover, it can also potentially be a requirement for initiating apoptosis, depending upon the threshold of oxidative and other stresses faced by the cells such as nutrient depravation, inflammation, etc. [40,51,52]. Therefore, it is possible that in the case of AOB (S) in HeLa cells, since efficient apoptosis can be induced most of which is reversible by Z-VAD-FMK, the effects on autophagic markers being observed are more of a “survival” response by the cell using autophagy, rather than a cell death mechanism. 

**Figure 10:**
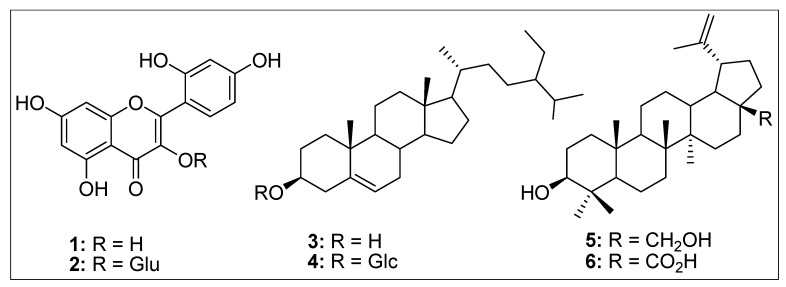
Structures of some the compounds isolated from *A. orientalis*. **1**: Morin, **2**: Morin-3-*O*-β-D-glucopyranoside, **3**: β-sitosterol, **4**: *β-*sitosterol-3-*O*-β-D-glucopyranoside, **5**: Betuline, and **6**: Betulinic acid.

Thus, *A. orientalis* is a strong candidate plant for the isolation of different types of anticancer compounds. The biologically active components of medicinal plants normally include flavonoids, phenolics, and polyphenols with promising anticancer activities [53,54]. We have previously shown that *A. orientalis* extracts/fractions contain substantial amounts of flavonoids and phenolic compounds with effective antioxidant, lipid peroxidation, and cytotoxic activities against colorectal and liver cancer cells, such as HT29, HCT116, and HepG2 [22]. A subset of these fractions was also shown to contain inhibitory potential against two enzymes important for sugar metabolism and survival of bacteria in the host; α-glucosidase and urease enzymes, respectively [22]. Furthermore, we have reported isolation of two different flavonoids with anti-proliferation activity against the same colorectal and liver cancer cells [20]. One of the isolated flavonoids, morin (Figure 10, structure 1), had a 40–50% inhibitory effect on these cell lines, while the other compound, morin-3-*O*-β-d glucopyranoside (Figure 10, structure 2), had a more drastic effect with 60–90% mortality of cancer cells [20]. These results have been supported by other studies where different groups have isolated and reported cytotoxic potential of morin flavonoids from several other species of plants in breast cancer cell lines, such as MCF-7 and MDA-MB-231 and many other cancer cell lines [55,56,57,58,59]. 

Other than compounds **1** and **2**, we have also identified four triterpenes from the low polar fraction (dichloromethane) of *A. orientalis*, including β-sitosterol (**3**), *β-*sitosterol-3-*O*-β-d-glucopyranoside (**4**), betuline (**5**), and betulinic acid (**6**) (Figure 10) [18]. These compounds have been screened by other investigators from different species for their cytotoxic potential against breast, cervical, and other cancer cell lines and in some cases mice with promising results [55,58,60,61,62,63,64,65,66,67,68,69,70,71,72]. Thus, these data suggest that *A. orientalis* is a medicinal plant enriched in phytochemicals with promising anticancer activity. 

## 4. Materials and Methods 

### 4.1. Cell Lines

The anticancer potential of the various organic extracts/fraction of *A. orientalis* was tested in four human cell lines, including: the normal breast epithelial cell line (MCF-10A), hormone responsive (MCF-7) cell line, triple negative (MDA-MB-231) cell line, and human cervical cancer cell line, (HeLa). The cells were cultured in ATCC-recommended media and maintained at 37 °C in a 5% CO_2_ incubator. Cells were plated a day before conducting the experiments and seeded at the cell density specified. 

### 4.2. Plant Collection

The air-dried whole plant material (8.2 kg) of *A. orientalis* was collected from different places of *Al-Hamra*, in *Ad-Dakhiliyah* region of the Oman (2012) and identified by a plant taxonomist at the Department of Biological Sciences and Chemistry, University of Nizwa, Nizwa, Oman [22]. 

### 4.3. Extraction and Fractionation

The extraction and fractionation of the leaf and stem parts of the plant has been described before [22]. Briefly, the powdered plant material of leaves (AO (L), 3.3 kg) and stem (AO (S), 4.1 kg) was initially soaked in methanol with vigorous shaking. The combined MeOH filtrates were evaporated under reduced pressure, resulting in a greenish crude extract of stem (AOM (S), 339.1 g) and leaves (AOM (L), 300 g), respectively. The MeOH extract of leaves was then partitioned into *n*-hexane (AOH (L), 40.9 g), dichloromethane (AOD (L), 84.8 g), ethyl acetate (AOEA (L), 30 g), *n*-butanol (AOB (L), 58.3 g), and aqueous (AOAQ (L), 76.7 g) fractions. The same procedure was carried out for the crude extract of stem residue to obtain *n*-hexane (AOH (S), 11.8 g), ethyl acetate (AOEA (S), 17.4 g), dichloromethane (AOD (S), 205.5 g), *n*-butanol (AOB (S), 13.4 g), and aqueous (AOAQ (S), 67.3 g) fractions.

### 4.4. Plant Extract/Fractions Preparation for Cytotoxicity Studies

Organic extracts/fractions from *A. orientalis* were solubilized in dimethyl sulfoxide (DMSO) except the aqueous fraction which was solubilized in Millipore water to prepare stock solutions at 12.5, 25, and 50 mg/mL concentrations, depending upon the form and solubility of fraction, and then stored at −80 °C. The stock solutions were used to make different extract treatments in cell culture media as needed. The DMSO control contained the same concentration of DMSO as present in the tested extracts/fractions as a control for cell death induced by DMSO alone. 

### 4.5. MTT Cell Viability Assay

Cells were cultured in 96-well plates at a density of 5 × 10³ cells/well/100 µL media. After 24 h, cells were treated with 100 µL of the different concentrations of the extracts/fractions at twice the final concentration, DMSO (as a negative control), or culture media alone for various time points. A stock solution of MTT [3-(4,5-dimethylthiazol-2-yl)-2-5-diphenyltetrazolium bromide] (5 mg/mL) was added at 25 µL/well and the plates were incubated for 3–4 h in a 37 °C incubator. The formazan crystals thus formed were dissolved by adding 200 µL of DMSO. Absorbance was measured at 560 nm. Cell viability was calculated as a percentage of cells treated with DMSO alone as a control using the same concentrations of DMSO. Results were analyzed using MS Excel and GraphPad Prism v5 software. 

### 4.6. Luminescent Cell Viability and Caspase-Glo^®^ 3/7, 8 and 9 Assays 

Appropriate cell lines were cultured in 96-well, opaque white plates (Thermo Fisher Scientific, Waltham, MA, USA) at a density of 5 × 10³ cells/well/100 µL, as described before [29]. After 24 h, 50 µL of the cell culture was removed and treated with 50 µL of the different extract/fraction concentrations at twice the final concentration. After 48 h of incubation, cells were assayed for cell viability using the Cell Titer-Glo Luminescent Cell Viability and Caspase-Glo^®^ 3/7, 8 and 9 Assays (Promega Corporation, Fitchburg, WI, USA) as per manufacturer’s directions. Luminescence was measured using the Infinite M200 Pro Tecan plate reader. Data were presented as percent cell viability of experimental groups relative to DMSO-treated cells taken as 100%. 

### 4.7. Western Blot Analysis 

Total cellular protein lysates (40 µg per lane) prepared in RIPA buffer (10 mM Tris-Cl (pH 8.0), 1 mM EDTA, 1% Triton X-100, 0.1% sodium deoxycholate, 0.1% SDS, 140 mM NaCl) were loaded onto 8–12% SDS-polyacrylamide mini gels (Bio-Rad) and blotted onto nitrocellulose membranes using standard protocol. After wet transfer, the membranes were blocked with 5% low fat dried milk in 1 x PBS and 0.1% Tween-20 (PBST) for 1 h. The membranes were then incubated at 40 °C overnight with primary antibodies in 1% milk in PBST purchased from Sigma-Aldrich, St. Louis, MO, USA (anti-actin; cat. no A-3854) or Cell Signaling Technologies (CST), USA as per manufacturer’s instructions. The list of the antibodies from CST includes: anti-PARP (cat. no. 9542) and anti-caspase 3, 7, 8, and 9 (cat. nos. 9668, 9492, 9746, and 9502, respectively). The blots were then incubated with the appropriate secondary antibodies (anti-mouse or anti-rabbit) for 1 h and 30 min at room temperature followed by detection using the Pierce™ ECL Plus Western Blotting Substrate and visualized using Typhoon reader. 

### 4.8. Flow Cytometry of Annexin V/Propidium Iodide-Stained Cells

Signs of early and late apoptosis were studied by analyzing the ability of the different fractions of *A. orientalis* methanol extracts to induce flipping of the phosphatidylserine phospholipid from the inner plasma membrane to the outer layer of the phospholipid bilayer. This was achieved by detection of phosphatidylserine by its affinity to annexin V which was fluorescently labelled and staining for live or dead cells by the propidium iodide vital dye using a kit from Becton Dickenson, (Franklin Lakes, NJ, USA). Stained cells were visualized by standard flow cytometry (BD FACS Canto II, Franklin Lakes, NJ, USA) where all cells counted (10,000 per sample) were included in the gating to prevent sample bias. Apoptosis was inhibited by the pretreatment of cells for two hours with 20 µM of Z-VAD-FMK (Promega Corporation, Fitchburg, WI, USA), while necroptosis was inhibited by pretreatment of cells with 50 µM Necrostatin-1 for 2 h (Sigma-Aldrich, St. Louis, MO, USA). 

### 4.9. Morphological Studies 

Cell cultures treated with extracts/fractions were followed for morphological changes by observation via an inverted light microscopic attached to a CCD camera or EVOS Cell Imaging System (Thermo Fisher Scientific, Waltham, MA, USA). The treated cells were compared with cells treated with similar DMSO concentrations and untreated cells. Images were saved at various magnifications at different times after treatment. 

### 4.10. Statistical Analysis

Statistical analysis of the presented data was conducted using either Microsoft Excel or Graphpad Prism v7 software. Average values were calculated for each sample in replicates of three or more followed by calculation of their standard deviations that were plotted as error bars on the column graphs. Paired, two-tailed student’s t-test was used to determine significant differences between any two groups. The extent of significance was shown as either one to three stars, depending upon the value obtained (* *p* < 0.05; ** *p* < 0.01 but >0.001; *** *p* < 0.001). The dose- and time-dependent Graphpad figures were generated by normalizing the values obtained for the test groups with that of their DMSO control since a specific DMSO control was used for each extract/fraction tested, whether it was the MTT assay or the Promega Glo assays. The LC_50_ values were calculated using Graphpad Prism software using non-linear regression and least square fit to get the values.

## 5. Conclusions

Characterization of the anticancer potential of *A. orientalis* has revealed that its leaves and stems harbor bioactive compounds that can inhibit cancer cell proliferation. However, the leaves have more potent anticancer compounds than the stems that primarily use non-apoptotic pathways for the induction of cell death. The anticancer bioactive compounds in the stem are less damaging to the normal cells and are more efficient at inducing caspase-dependent apoptosis. Both the leaf and stem fraction could trigger necroptosis as well, though the stem fraction could do it at lower concentrations only. Similarly, both fractions led to the perturbation of autophagy in HeLa cells, but in a different manner. Together, we conclude that *A. orientalis* is a valuable medicinal plant for the search of novel anticancer agents with variable biological activities for different types of cancer cells required to fight the battle against cancer. 

## Figures and Tables

**Table 1:** First screening of *A. orientalis* leaf (L) and stem (S) extracts/fractions for their antiproliferative effects on cancer cells using the MTT assay.

Extracts/Fractions	MCF-7 (µg/mL) Breast Cancer Cells	HeLa (µg/mL) Cervical Cancer Cells
AOH (L)	X	X
AOD (L)	√ (250; 72 h)	√ (125; 72 h/250; 72 h)
AOEA (L)	X	√ (125; 72 h/250; 72 h)
AOB (L)	X	X
AOM (L)	X	X
AOAQ (L)	X	X
AOH (S)	X	X
AOD (S)	X	√ (125; 72 h)
AOEA (S)	X	X
AOB (S)	X	√ (250; 72 h)
AOM (S)	X	X
AOAQ (S)	X	X

X = Extracts/fractions not effective for inhibiting cell proliferation (≤20% inhibition) of either cell line; √ = Extracts/fractions which resulted in >20% inhibition of cell proliferation; Abbreviations: *A. orientalis* (AO) leaf (L) or stem (S) fractions using different organic solvents; AOH = *n*-hexane; AOD = dichloromethane; AOEA = ethyl acetate; AOB = *n*-butanol; AOM = methanol; AOAQ = aqueous fraction.

## Abbreviations

DMEM	Dulbecco’s Modified Eagles Medium
DMSO	Dimethyl Sulfoxide
FBS	Fetal Bovine Serum
EGF	Epidermal Growth Factor
FCS	Fetal Calf Serum
MTT	[3-(4,5-dimethylthiazol-2-yl)-2-5-diphenyltetrazolium bromide]
PARP	Poly (ADP-ribose) Polymerase
PBS	Phosphate Buffered Saline
PBST	Phosphate Buffered Saline with 0.1% Tween
PMSF	Phenylmethyl Sulfonyl Fluoride
RIPA	Radioimmunoprecipitation Assay Buffer
SDS	Sodium Dodecyl Sulfate
